# Supplementary material for: Learning curve and surgical outcome of robotic assisted colorectal surgery with ERAS program
Source: Sci Rep. 2022 Nov 29;12:20566. doi: 10.1038/s41598-022-24665-w (PMC9709162; doi:10.1038/s41598-022-24665-w)
Supplement: Supplementary file 2 — Supplementary Information 2. [file 41598_2022_24665_MOESM2_ESM.zip › RAL Raw data-Ñ[▒K/137/HS_ñw╝╨░O▒Kñσ.pdf]

正式摘要(電子簽章：000000233509000)		
W95 / W95 ☐英文標題(注意:無英文姓名-請補病人基本檔!)	☐【讀取】憑證	

臺中榮民總醫院

出院病歷摘要

轉入號 : 轉出號 :

[redacted]

(8)流水編號	
(9)入院日期	110/12/29 (2021) 直腸外科 W95 - 05
(10)轉科日期	
(11)出院日期	111/01/03 (2022) 住院天數計 5 日

診斷

- 入院 :1. Rectum cancer
  2. HCVD under medication control
  3. Anal fistula s/p OP
- 出院 :1. Rectum cancer s/p Robotic assist LAR + Loop ileostomy on 2021/12/30
  2. HCVD under medication control
  3. Anal fistula s/p OP
  4. Newly diagnosed DM

主訴

- Bloody stool off and on

病史

- This is a 76 years old male patient with history of

  1. HCVD under medication control

  2. Anal fistula s/p OP

  According to the previous medical record, he suffered from bloody stool off and on for 1 months. He visit our CRS OPD, and colonoscopy with biopsy was done on 2021/12/06, showed adenocarcinoma over rectum, 10cm FAV. Under the impression of Rectum cancer, this time he was admitted to our ward for scheduled Robotic assist LAR.

體檢發現

- DENIED ABD OP HXM BUT ANAL FISTULECTOMY 30 YEARS AGO ....
  - General appearance :
    - easy looking , conscious clear , without cardiopulmonary distress
    - Vital signs自動帶入date 體溫(BT) 脈搏(P) 呼吸(R) 血壓(BP) 身高(BH) 體重(BW) BMI
      20211229 36℃ 78次/分 18次/分 160/78mmHg 156.5cm 68.4kg 27.9
    - Vital signs: B.P. 160/78 mmHg , P.R. 78 /min , R.R. 18 /min , B.T. 36 ℃
  - HEENT :
    - Eyes: no anemic conjunctiva, no icteric sclera, normal EOM
    - ENT: no otorrhea, no rhinorrhea, no erythematous mucosa, no injected tonsil, no deformity
  - Neck: supple, freely movable, trachea midline,no deformity, no engorged jugular vein , no palpable thyroid or lymph node enlargement
  - Chest:
    - Inspection: Grossly normal, symmetric and fully expansion, no accessory muscle used, no operation scar, no spider angiomata, no gynecomastia
    - Palpation: no palpable mass or nodule, no subcutaneous emphysema, no tenderness
    - Ausculation: clear breathing sound
  - Heart:
    - Palpation: no thrill, no heave
    - Percussion: normal heart size

- ○ Ausculation: regular heart beat, normal S1, S2, no gallop, no murmur
- Extremities: No deformity, full ranged of motion without limitation, no clubbing finger or nail cyanosis, no pitting edema, no ulcer or pigmentation, no obvious skin lesion, no varicose vein, no bone, joint or muscle tenderness

CRS CONDITION

- Karnofsky performance status: (100) Normal, no complaints, no evidence of disease
- PE of Abdomen:
- CEA: 2.66 ng/ml
- Imaging Studies:
- Colonoscope report: .
- Figures:
  1. colon & rectum

手術日期及方法

- 手術日期及方法20211230 1. Robotic (XI) assisted low anterior resection
  2. Intraoperative colonoscopy : (-).
  3. High ligation (+) : At IMA root (Via robotic clips). IMV was ligated via endoclip.
  4. Take down from the splenic flexure : (-)
  5. Robotic EndoGIA (45-4.3) X3 + EEA (29mm) + OpenGIA (80-3.8) X1 were applied to complete the side-to-end anastomosis
  6. Proximal cut end: > 10 cm, distal cut end: 3 cm
  7. No drainage tube was inserted
  8. ADHESION BARRIER FILM was placed
  9. Loop ileostomy (About 15~20cm from IC valve) was done over RLQ of abdomen
- 手術結果及發現 :
  - 手術日期: 1101230 麻醉方式:
    手術名稱:1. Robotic (XI) assisted low anterior resection 2. Intraoperative colonoscopy : (-). 3. High ligation (+) : At IMA root (Via robotic clips). IMV was ligated via endoclip. 4. Take down from the splenic flexure : (-) 5. Robotic EndoGIA (45-4.3) X3 + EEA (29mm) + OpenGIA (80-3.8) X1 were applied to complete the side-to-end anastomosis 6. Proximal cut end: > 10 cm, distal cut end: 3 cm 7. No drainage tube was inserted 8. ADHESION BARRIER FILM was placed 9. Loop ileostomy (About 15~20cm from IC valve) was done over RLQ of abdomen
    術前診斷:RECTAL CA 10CM FAV
    術後診斷: Ditto.s/p robotic LAR+ loop ileostomy

住院治療經過

- After admission, he received Robotic assist LAR + loop ileostomy on 2021/12/30. He start clear liquid diet on 12/31 and try bland II diet on 1/01. CVC and ROD are removed on 1/03, and we consult CV for HTN, suggest add Carvedilol 25mg BID. Under relative stabel condition, he discharged on 1/03, OPD follow up.

合併症

一般檢查

- 累積報告 :

DATE	TIME	ALB	TP	BIL.T	BIL.D	ALKP	AST	ALT	LDH	AMY	LIPASE	NH3	Ferritin	UA
1101229	1723	4.0	6.5	0.8	0.2	109	24	37						5.5
1101230	1550	3.4		1.15	0.30		28	38						

DATE	TIME	WBC	RBC	HGB	HCT	MCV	PLT	BLST	NEUT	BAND	SEG	LYM	MON	EOS	BAS
1101229	1722	12100	4.90	15.0	45.0	91.8	288		80.4			15.0	4.0	0.2	0.4
1101230	1550	19980	4.57	13.9	41.1	89.9	235		90.8			4.2	4.8	0.0	0.2

DATE	TIME	NA	K	CL	BUN	CREAT	eGFR	CA	Mg	P	CRP	ESR	PCT	D-Dimer	LACTATE
1101229	1723	138	3.2		19	0.90	87.2	8.2			0.047				
1101230	1550	138	3.2	102	19	1.19		8.7							

DATE	TIME	GLUCOSE	HbA1C	CHOL	TG	HDL	LDL	KETONE	CK	CKMB	TRO-T	PT-P	APTT-P	NT-pro BNP
1101229	1723	114		217	110	49	146							

特殊檢查

病理報告

其它

出院時情況

- 3.改門診治療

出院指示

-

藥名	劑量	劑量單位	用法	頻次	數量	數量單位	現況	天數	單位
Dilatrend tab 25mg	1	TAB	PO	BID	16	TAB	TH	8	天
Ultracet tab	1	TAB	PO	Q6HPRN	32	TAB	TH	8	天
Cozaar tab 50mg	1	TAB	PO	QD	8	TAB	TH	8	天
Norvasc tab 5mg	1	TAB	PO	BID	16	TAB	TH	8	天
(□)Takepron OD tab 30mg	1	TAB	PO	QDAC	8	TAB	TH	8	天
Suzole eye drops 4% 5mL		BOT	OU	QIDPRN	0	BOT	TH	1	瓶

出院計劃

- 門診日期
  - date on 2022/01/11

主治醫師 2062F 蔣鋒帆 / 住院醫師 2339C 蔡濬樟

正式摘要(電子簽章：000000233509000)

W95 / W95

doctorRank:B
